# Imaging manifestations and treatment efficacy of Granulomatous Lobular Mastitis: An analysis of 29 cases with internal and external traditional Chinese medicine approaches

**DOI:** 10.12669/pjms.40.10.8981

**Published:** 2024-11

**Authors:** Qiuping Ning, Zheng Zhang, Hua Ren, Tiebing Fan, Chunzhi Li

**Affiliations:** 1 Qiuping Ning, Department of Radiology, Kulun Banner Mongolian Medicine Hospital, Tongliao City, Inner Mongolia 028200, China. Department of Radiology, China Academy of Chinese Medical Sciences, Xiyuan Hospital, Beijing 100091, China; 2 Zheng Zhang, Department of Radiology, Kulun Banner Mongolian Medicine Hospital, Tongliao City, Inner Mongolia 028200, China; 3 Hua Ren, Department of Radiology, China Academy of Chinese Medical Sciences, Xiyuan Hospital, Beijing 100091, China; 4 Tiebing Fan, Postdoctoral Management Office, Chinese Academy of Chinese Medical Sciences, Beijing100027, China; 5 Chunzhi Li, Department of Radiology, China Academy of Chinese Medical Sciences, Xiyuan Hospital, Beijing 100091, China

**Keywords:** Granulomatous lobular mastitis, Abscess, Diffusion magnetic resonance imaging, Image Enhancement, Chinese traditional, Medicine

## Abstract

**Objectives:**

Using magnetic resonance imaging (MRI), we analysed the clinical manifestations of granulomatous lobular mastitis (GLM) before and after treatment with traditional Chinese medicine (TCM).

**Methods:**

This was a retrospective study. Clinical manifestations and imaging features of patients with biopsy-proven GLM before and after treatment were retrospectively analysed from April 2021 to April 2023 at Xiyuan Hospital, China Academy of Chinese Medical Sciences.

**Results:**

Among 29 women of childbearing age (mean age, 34.3±3.6 years), Compared to pre-therapy, the number of patients with lumps, ruptures, pain, and menstrual irregularities was significantly reduced (P < 0.05). The Chinese medicine staging demonstrated a transition from bulk to ulcerated (P=0.041). Pre- and post-treatment MRI changes included lesion number, distribution, maximal diameter, time-signal intensity curves (TIC), apparent diffusion coefficients (ADC), and BI-RADS categories 3 and 4a (all P<0.05). Furthermore, substantial changes were seen in chest wall invasion, nipple discharge, increased feeding arteries, and skin edema (all P<0.05).

**Conclusion:**

Combining internal and external Traditional Chinese Medicine (TCM) therapy proved successful for GLM. A reliable assessment of therapy efficacy is provided by MRI examination of lesion changes before and after treatment.

## INTRODUCTION

Women of childbearing age are commonly affected by granulomatous lobular mastitis (GLM), a benign chronic disease of the breast of unknown aetiology.^1^ The International GLM Consensus, published in 2021, strongly advocates Magnetic resonance imaging (MRI) for detecting active lesions, assessing lesion scope, postoperative recurrence, or monitoring conservative treatment.^2^ Contrast-enhanced (CE) MRI tracks changes in breast lesions, aiding in distinguishing cancer and healthy breast tissue.^3^ Diffusion-weighted imaging (DWI) has the potential to be effective in tumour staging and can reveal histological characteristics of various lesions.^4^,^5^ Previous research in GLM has highlighted non-mass clustered annular enhancement and diffusion-limited MRI findings.^6^

GLM is typically treated with conservative observation, antibiotics, corticosteroids, partial or major excision, and mastectomy,^1^,^7^ it has also been manged through Traditional Chinese Medicine (TCM).^8^ With limited studies on MRI evaluations of GLM efficacy, our retrospective study examined clinical and MRI data before and after TCM treatment. We aimed to assess the effectiveness of internal and external TCM treatments for GLM diagnosis and management.

## METHODS

This was a retrospective study. Twenty-nine patients with pathologically confirmed granulomatous lobular mastitis (GLM) who underwent MRI examinations between April 2021 and April 2023. All cases were histologically verified. We reviewed patient records to gather data on demographics, clinical presentations, and imaging characteristics. The imaging features of the lesions were analysed ultrasound (US), and magnetic resonance imaging (MRI). Images were retrieved from our PACS and analysed retrospectively. Patients at the time of the first MRI examination averaged (34.3 ± 3.6) years (range: 28-40 years), with a duration ranging from 1 to 26 m (average: 5.5 m).

### Ethical Approval:

The China Academy of Chinese Medical Sciences Ethics Review Committee approved this retrospective study at Xiyuan Hospital (No.: 2022XLA010-1; date: 2023-01-25). Owing to the retrospective nature of the study, informed consent from the patients was not required.

### Inclusion criteria:

- Patients diagnosed with GLM confirmed by biopsy;
- Who have complete preoperative MRI examination data.

### Exclusion criteria:

- Patients who have undergone surgery followed by radiotherapy, or targeted therapy;
- Those with contraindications for MRI examination.

Various treatments were administered before admission, including conservative (four), antibiotic (six), hormone and methotrexate (ten), and TCM (twelve). The formula was based on Xiaoyao Sanjie Decoction,^9^ and was adjusted based on the formula and administered externally with heated medicinal residue according to the different symptoms of the patients at different stages. Breast MRIs were repeated one month after treatment, and demographic and imaging data were analysed retrospectively. Owing to the retrospective nature of the study, informed consent from the patients was not required.

Scans were performed using a 3.0T MRI scanner (Discovery 750; GE Healthcare, Waukesha, WI, USA). Patients underwent DWI and CEMRI, employing a spin-echo sequence with a TR of 3125 ms, TE of 84 ms, and b-values of 0s/mm^2^ and 1000s/mm^2^. At an injection rate of 3.0 ml/s, gadodiamide was injected into the elbow vein cluster at 0.1 mmol/kg and followed by saline flushing for CE-MRI scan. The second six images were automatically clipped with the first image of the observed subject.

**Fig.1:**
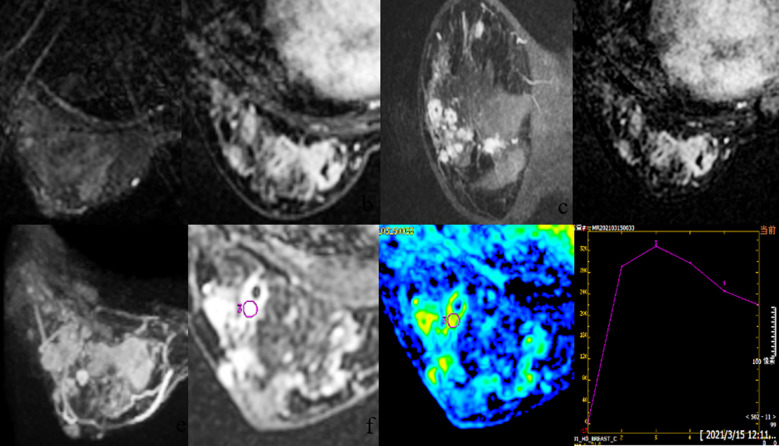
A35-year-old lady had “breast hyperplasia” with dilated mammary ducts (BI-RADS level 4a) On ultrasonography. MRI revealed numerous irregular cystic nodules in the left breast, one of which was approximately 2.5 cm in diameter, isointensity on T2WI (1a), heterogeneous strong signal on DWI (1f), and ADC 2.08 10-3 mm2/s (1 g). Axial contrastenhanced picture of a breast with clustered annular enhancement (1b-c), and the silhouette highlighting the lesion (1d). (MIP image 1e) indicates increased edema of the left breast surface as well as thickening of the blood supply artery. GLM findings were verified by pathological findings (left breast).

The images were evaluated independently by two radiologists with five and eight years of breast imaging experience. Disagreements were resolved through joint discussion. Scans were conducted using a GE workstation (Advantage Windows 4.5, General Electric, Madison, WI, USA) and Functool 4.4 software. Breast Imaging Reporting and Data System (BI-RADS)^10^ was used to evaluate the lesion based on the analysis of the distribution, signal intensity, size, and enhancement pattern of the lesion after contrast injection. Time-signal intensity curves (TIC) were plotted following KUHL staging criteria.^11^ Apparent diffusion coefficient (ADC) was calculated from a region of interest (ROI) excluding necrotic cystic areas, determined from the lesion’s largest dimension on DWI. (Fig.1). Additional findings like abscess, sinus or fistula formation, nipple, skin oedema, and enlarged axillary lymph nodes, were observed and counted. A comparison and analysis of MRI images were conducted before and after the treatment.

### Statistical Analysis

Statistical analyses were conducted using SPSS 22. 0 software (SPSS Inc., Chicago, IL, USA) and the *Shapiro-Wilk* test. If quantitative data were normally distributed, a *t*-test was applied. An independent samples *t*-test was used to compare the differences in continuous data between the two groups,. For non-normally distributed data, a rank-sum test was performed. To compare qualitative data, the χ^2^ test and Fisher’s exact test were used. Statistical significance was set at p< 0.05.

## RESULTS

All patients were women of childbearing age, with the majority (51.7%) had a pregnancy history within the past five years (17.2%) had used contraceptives. Table-I. A family history of breast cancer was present in four cases (13.8%), and obesity in three cases (10.3%). Seven cases (24.1%) were associated with hyperprolactinemia.

**Table-I:** Demographic and Clinical Characteristics of Patients with GLM (N=29)

Characteristics	GLM (n=29)
Age, mean (SD)	34.3 (3.6)
Duration of illness, (m), (median)	1-26 (5.5)
***Menopausal status, n (%)***	
Reproductive age, regular	17 (58.6)
Reproductive age, irregular	12 (41.4)
Perimenopausal, irregular	0 (0)
Post-menopausal (natural/post-operative)	0 (0)
***Pregnancy status (last delivery to date), n (%)***	
Current Pregnancy	0
<1 year	3 (10.3)
1-5 years	15 (51.7)
>5 years	8 (27.6)
No delivery	2 (6.9)
Unknown	1 (3.5)
***Hormonal contraceptive use, n (%)***	
Current	5 (17.2)
None	24 (82.8)
Other, n (%)	
Family history of breast cancer (immediate family)	4 (13.8)
Hyperprolactinemia	7 (24.1)

***Notes:*** SD: standard deviation; BMI: body mass index. *Others include Mongolian, Yi: Tibetan, and other ethnic minority groups.

After treatment, the incidences of lumps, rupture, pain, and irregular menstruation were significantly lower than before (all P<0.05) (Table-II). Significant variations in nodular masses, pressure pain, erythema, and skin fistulas were also observed before and after treatment (P<0.05). Traditional Chinese Medicine (TCM) classified GLM stages as mass, abscess, and late-stage ulceration. 8 Initially, the mass stage predominated (44.8%), but after therapy, the prevalence of late-stage ulceration increased considerably (55.2%, P=0.041).

**Table-II:** Clinical Characteristics Before and After TCM Treatment (N=29)

Characteristic	Before treatment (n=29)	After treatment (n=29)	P-value
***Clinical presentation, n (%)***			
Lumps	26 (89.7)	11 (37.9)	<0.001
Rupture	24 (82.8)	5 (17.2)	<0.001
Nipple discharge	5 (17.2)	0	1.0
Pain, pressure pain	13 (44.8)	2 (6.9)	0.001
Menstrual irregularity	26 (89.7)	8 (27.6)	<0.001
***Physical examination***			
Nodular, lumps	26 (89.7)	12 (41.4)	0.0001
Pain, pressure pain	27 (93.1)	8 (27.6)	<0.001
Erythema	15 (51.7)	5 (17.2)	0.0057
Skin fistula	19 (65.5)	3 (10.3)	<0.001
Orange peel-like appearance, armour	4 (13.8)	3 (10.3)	1.000
Nipple discharge	20 (68.9)	14 (48.3)	0.1097
Inverted nipples	4 (13.8)	4 (13.8)	0.7034
***TCM clinical staging, n (%)***			1.000
Mass stage	13 (44.8)	5 (17.2)	
Abscess stage	8 (27.6)	3 (10.3)	
Late stages of ulceration	8 (27.6)	3 (10.3)	

***Note:*** TCM: Traditional Chinese Medicine.

Multiple nodules were common prior to therapy (79.3%) (Table-III), but their prevalence considerably decreased afterward to single lesions (69.0%, P=0.0216). Segmental (55.2%) to scattered (41.4%) lesion distribution changed, albeit this change was not statistically significant (P=0.057). Prior to and following therapy, mammary duct dilatation remained stable (48.3% vs. 69.0%, P=0.271). Post-treatment, inhomogeneous enhancement patterns grew (69.0% vs. 41.4%, P=0.056). Treatment considerably changed the enhancement curves (P=0.057), increasing the ADC values from (1.19±0.35) 10^-3^ mm^2^/s to (1.57±0.68) 10^-3^ mm^2^/s (P=0.0098). Post-treatment, BI-RADS category 3 classification decreased (96.5% vs. 86.2%, P<0.001), with category 4a tumours showing the greatest decline (3.5% vs. 10.3%, P<0.001). There was no discernible difference in the size of the ipsilateral axillary lymph nodes (48.3% vs. 65.5%, P=0.523). Chest wall invasion, nipple discharge, swollen feeding arteries, and skin edema all showed significant improvements (P<0.001, 0.0002, 0.0164 and 0.0022, respectively).

**Table-III:** MRI Performance Before and After TCM Treatment (N=29)

Characteristic	Before TCM treatment (n=29)	After TCM treatment (n=29)	P-value
Number of nodules, n (%)			0.0216
Individual	6 (20.7)	9 (31.0)	
Number of nodules (more than 2)	23 (79.3)	20 (69.0)	
Distribution, n (%)			0.057
Segmental distribution	16 (55.2)	8 (27.6)	
Local focal distribution	3 (10.3)	9 (31.0)	
Decentralised distribution	10 (34.5)	12 (41.4)	
Dilated mammary ducts, n (%)			0.271
Unilateral	12 (41.4)	7 (24.1)	
None	14 (48.3)	20 (69.0)	
Bilateral	3 (10.3)	2 (6.9)	
Maximum diameter of lesion (cm), mean (SD)	3.9 (1.9)	1.3 (0.9)	<0.001
Enhancement patterns, n (%)			0.056
Heterogeneous	12 (41.4)	20 (69.0)	
Clustered annular	9 (31.0)	7 (24.1)	
Uniformity	8 (27.6)	2 (6.9)	
TIC, n (%)			0.028
Type II	6 (20.7)	15 (51.7)	
Type III	23 (79.3)	14 (48.3)	
ADC, mean (SD) (10-3 mm2 /s)	1.19 (0.35)	1.57 (0.68)	0.0098
BI-RADS classification, n (%)			
category 3	25 (86.2)	28 (96.5)	<0.001
category 4a	3 (10.3)	1 (3.5)	<0.001
category 4b	1 (3.5)	0	1.0
category 4c	-	-	
category 5	-	-	
category 6	-	-	
Enlarged axillary lymph nodes, n (%)			0.523
same side	14 (48.3)	19 (65.5)	
Bilateral	3 (10.3)	2 (6.9)	
No enlargement	12 (41.4)	8 (27.6)	
Other performance, n (%)			
Chest wall invasion	3 (10.3)	2 (6.9)	<0.001
Nipple discharge	6 (20.7)	3 (10.3)	0.0002
Inverted nipples	7 (24.1)	7 (24.1)	1.000
Enlarged feeding arteries	23 (79.3)	19 (65.5)	0.0164
Edema of the skin	9 (31.0)	4 (13.8)	0.0022

***Notes:*** SD: standard deviation; TIC: time-intensity curve; ADC: apparent diffusion coefficient. BI-RADS: Breast Imaging Reporting and Data System.

## DISCUSSION

The pre-treatment examination in this study was generally consistent, but the proportion was higher than that in the previous study,^12^ with 89.7% of the patients presenting with breast lumps. This may be due to the small sample size and the large number of patients in the mass stage of this study. Research shows that the proportions of patients with inverted nipples and nipple discharge were 17.7% and 15.6%,^12^ respectively and the proportion of patients with inverted nipples was 17.2% in this study, which is generally consistent with this, but the proportion of patients with nipple charge was higher, considering that related to the duration of illness, and the size of the lesion.

In line with previous reports,^13^ patients in this study were women of reproductive age, and five had taken hormonal contraceptives in the past. Several factors are believed to contribute to the pathogenesis of GLM, including excessive levels of oestrogen and progesterone (during pregnancy or ingestion), elevated prolactin levels and abnormal hormone levels in the body.^14^

In this investigation, GLM showed heterogeneous, clustered annular enhancement resembling granulomatous hyperplasia and microabscesses, with central necrosis resembling ductal cancer,^7^ which is similar to previous study (Aslan et al.^6^). Diagnosis involved considering clinical symptoms, maternal history, and histopathology.^6^

The initial ADC values ((1.19 ± 0.35) × 10^–3^ mm^2^/s) were slightly lower than reported in earlier investigations (1.244 ± 0.32) × 10^-3^mm^2^/s), 14 attributed to factors like inflammatory cell infiltration and tissue changes in GLM.^15^ Researchers recommend that a typical GLM be classified according to BI-RADS category four, for atypical GLM, it is recommended to category of five.^16^ There is evidence that 1.4%-12.5% of ductal carcinomas in situ of the breast have anterior lymph node metastasis.^17^ Therefore, lymph node enlargement in GLM patients cannot be ruled out the likelihood of breast cancer, this study identified 17 patients with enlarged axillary lymph nodes. Malignant morphology demanded attention, whereas benign looks necessitated a biopsy to rule out malignancy.^18^

Compared to CEMRI, DWI offers early treatment insights, with ADC values indicating therapy outcomes.^19^ This study demonstrates that higher ADC can be a quantitative reflection of therapy success to some extent. Additionally, it shows how the disease can be successfully treated with TCM’s internal and external remedies.

There is no optimal treatment for GLM at this time, and issues including side effects, recurrence, postoperative breast deformity, and psychological trauma are difficult to address.^20^ Clinical investigations have indicated that conservative GLM treatment with Chinese medicine has an 85% cure rate.^21^ The main etiological processes of GLM have been postulated to be spleen insufficiency and phlegm turbidity.^9^ Years of clinical and research investigations have demonstrated the efficiency and safety of Xiaoyao Sanjie Decoction,^22^ in improving wound healing, minimizing granulation, and enhancing immunity. This approach led to reduced chest wall invasion, smaller and fewer nodules, highlighting its effectiveness. A reduction in the number of nodules and size, and a reduction in chest wall invasion are some of the benefits of this treatment. The treatment with Chinese herbal medicine greatly reduced the number of patients in the bulk stage. If any suspicious findings are identified during imaging or clinical examination, histopathological analysis should be carried out. 23

### Limitations:

While this study demonstrated the efficacy of GLM imaging and TCM, not all GLM patients had access to or completed retrospective radiological tests. In other circumstances, data gaps may have resulted from limited retrospective access and varying examination occurrences. The retrospective nature also has drawbacks, which could lead to incorrect interpretations of radiological findings.

## CONCLUSIONS

Traditional Chinese Medicine effectively reduces inflammation and alleviates symptoms in GLM. Further research is crucial to confirm and expand these findings. Besides conventional breast imaging, magnetic resonance imaging helps in differential diagnosis and follow-up of therapeutic efficacy, but biopsy remains the definitive method for diagnosis.

### Authors’ Contributions:

**QN and CL** carried out the studies, data collection, drafted the manuscript, and are responsible and accountable for the accuracy or integrity of the work.

**HR and TF** performed the statistical analysis and participated in its design and review.

**ZZ** performed the statistical analysis and participated in its design.

All authors read and approved the final manuscript.
